# The safety of endothelin receptor antagonists in the treatment of pulmonary arterial hypertension: Protocol for a systemic review and network meta-analysis

**DOI:** 10.1097/MD.0000000000010122

**Published:** 2018-03-16

**Authors:** Zhi-Chun Gu, Yi-Jing Zhang, Mang-Mang Pan, Chi Zhang, Xiao-Yan Liu, An-Hua Wei, Ying-Jie Su

**Affiliations:** a Department of Pharmacy, Renji Hospital, School of Medicine, Shanghai Jiaotong University; b Department of Pharmacy, Third Affiliated Hospital of Second Military Medical University, Shanghai; c Department of Pharmacy, Tongji Hospital, Tongji Medical College, Huazhong University of Science and Technology, Wuhan, China.

**Keywords:** drug safety, endothelin receptor antagonists, pulmonary arterial hypertension, systematic review

## Abstract

**Background:**

Pulmonary arterial hypertension (PAH) is a progressive disease and ultimately leads to right heart failure. Endothelin receptor antagonists (ERAs) have been demonstrated to significantly improve prognosis in PAH. However, ERAs-induced side effects can result in poor patient tolerance. Thus, we aim to evaluate current safety evidence of ERAs in PAH.

**Methods:**

An electronic search will be performed for randomized controlled trials (RCTs) that reported the interested safety data (abnormal liver function, peripheral edema, and anemia) of ERAs in PAH. Risk ratios (RRs) with their confidence intervals (CIs) and the surface under the cumulative ranking curve (SUCRA) will be calculated using a network analysis.

**Results:**

This study will provide the safety evidence of ERAs in PAH by combining the results of individual studies based on direct- and network comparison, and to rank ERAs in the evidence network.

**Conclusions:**

The results will supplement missing evidence of head-to-head comparisons between different ERAs and guide both clinical decision-making and future research.

## 1 Introduction

Pulmonary arterial hypertension (PAH) is a life-threatening disease characterized by increasing pulmonary vascular resistance and pulmonary artery pressure, ultimately progressing to right heart failure and premature death.^[1]^ Drugs for PAH therapy, targeting the endothelial dysfunction and specific aberrant pathways, was approved by the US Food and Drug Administration.^[2]^ Currently, 5 classes of drugs was applied for PAH, including endothelin receptor antagonists (ERAs), prostanoids, phosphodiesterase type 5 inhibitors, soluble guanylate cyclase stimulators, and selective prostacyclin receptor agonists.^[2]^ Regarding ERAs, until now, 4 ERAs (bosentan, sitaxsentan, ambrisentan, and macitentan), which exert vasodilator and antiproliferative effects by binding to endothelin receptor type A (ETA) and/or B (ETB) in pulmonary vascular smooth muscle cells, have been demonstrated to significantly improve exercise capacity, symptoms, hemodynamics, and to slow clinical worsening in clinical trial.^[3–6]^ Nevertheless, along with their widespread clinical use, the safety of ERAs was gradually reported.^[7–9]^ Sitaxsentan, the first selective ERA antagonist, was withdrawn from the market worldwide in 2010 due to several reports of fatal liver injury in PAH patients.^[10]^ Abnormal liver function, peripheral edema, and anemia have been reported as the main adverse effects of ERAs in previous study. However, most of these studies included relatively small samples, and each study has reported a small number of adverse events. In addition, no head-to-head comparisons were addressed to assess the safety of ERAs in PAH. To boost precision results for decision-making, we aim to evaluate current safety evidence of ERAs in PAH by combining the results of individual studies based on direct- and network comparison, and to rank ERAs in the evidence network.

## 2 Methods

### 2.1 Data sources and searches

This systematic review and network analysis will be reported in accordance with standards outlined in the Cochrane Handbook and the PRISMA Extension Statement.^[11–13]^ A comprehensive literature search of Medline, Embase, and Cochrane Library electronic databases will be conducted to identify all potential eligible trials. Additionally, unpublished trails will be identified from the ClinicalTrials.gov Website. The bibliographies of published trials and systematic reviews will also be scrutinized to ensure that all relevant studies were identified. Two reviewers (ZCG and YJZ) will search the databases independently, and all disagreements will be resolved by consulting a third author (AHW).

### 2.2 Study selection

Studies will be included if they met the following criteria. The study design had to be a randomized controlled trial (RCT), and the population had to include adult patients with PAH. In addition, treatment had to include ERAs (bosentan, ambrisentan, or macitentan) and reported the interested safety data (abnormal liver function, peripheral edema, anemia) for ERAs and placebo separately. Two reviewers (ZCG and YJZ) will assess all study titles and abstracts, and full paper will be identified for any relevant possibility according to the inclusion. For reducing bias, ZCG and YJZ will be blinded to journal, authors’ names, and year of publication of the papers. All uncertainties and discrepancies will be resolved by consulting a third author (AHW).

### 2.3 Data extraction

Data will be extracted independently using a standard form, including study population characteristics (the name of the first author, publication year, sample size, mean age, sex, World Health Organization functional class, and etiology of PAH), treatment groups, comparison groups, baseline therapy, study duration, and all interested outcomes. Outcomes that were not reported in the publications will be further extracted from the ClinicalTrials.gov Website. Disagreements will be resolved by consensus after discussion.

### 2.4 Quality evaluation

The methodological quality of selected RCTs will be assessed employing the Cochrane Collaboration Risk of Bias Tool.^[14]^ The overall risk of bias will be determined as low (all items were low risk, or at least 5 items were low risk and the remaining 2 unclear), unclear (>2 items were unclear risk), and high (≥1 quality dimension suggested high bias).^[11]^

### 2.5 Bias assessment

Potential publication bias will be assessed by visually inspecting funnel plots, and will be minor if the plot of the magnitude of treatment effect in each study versus its precision estimate showed an approximate symmetrical funnel shape.^[12]^

## 3 Data analysis

We will use a network meta-analysis (NMA) by STATA software (version13, Statacorp, College Station, Texas) to carry out the direct and indirect comparison of treatments. Results will be reported as risk ratios (RRs) with their 95% confidence intervals (CIs). Heterogeneity, defined as variation beyond chance, will be evaluated through the *I*^2^ test when at least 2 studies are available for each pairwise comparison.^[12]^ For each direct comparison, a fixed-effect model will be performed unless *I*^2^ >50%. For inconsistency, we will use a node-splitting analysis to evaluate whether direct and indirect evidence on the split node is in agreement.^[15]^ For ranking, the surface under the cumulative ranking curve (SUCRA) will be employed to provide a hierarchy of the treatments. In our study, the larger SUCRA value is considered as the higher risk of the treatment. Moreover, sensitivity analyses will be conducted to identify the effect by excluding RCTs that combined with other PAH-specific drugs in baseline therapy. Statistical significance is set at a *P*-value <.05, and all tests performed are 2-sided.

## 4 Discussion

PAH is a progressive disease and ultimately lead to right heart failure.^[1]^ Liver damage, edema, and anemia might be the indication of right cardiac failure and worsening PAH.^[11]^ In clinical practice, it is difficult to distinguish the various etiologies of these clinical adverse effects in PAH patients. These adverse effects could occur due to worsening right-sided heart function, coadministrated drug side effects, or inadequate diuretic treatment.^[3–6]^ Even if the efficacy of ERAs is maintained, the development of these ERAs-induced side effects can result in poor patient tolerance.^[7,8]^ The present systematic review, based on network analysis, will pool current safety evidence of ERAs in PAH patients. It can supplement missing evidence of head-to-head comparisons between different ERAs and guide both clinical decision-making and future research. In the clinical setting, different monitoring parameters may be considered for different ERAs in PAH. Several possible limitations are worth mentioning. Firstly, we may not access to data according to various etiology of PAH or World Health Organization functional class, making statistical powerful subgroup analysis unavailable. Secondly, different baseline therapy may influence our analysis results. Thirdly, the observation time of included clinical trials may inconsistent, which might also influence our results. Furthermore, to our best knowledge, none of PAH studies have especially to be designed for assessing the safety of ERAs.
